# Protective Effects of *N*-Acetyl-L-Cysteine in Human Oligodendrocyte Progenitor Cells and Restoration of Motor Function in Neonatal Rats with Hypoxic-Ischemic Encephalopathy

**DOI:** 10.1155/2015/764251

**Published:** 2015-03-31

**Authors:** Dongsun Park, Kyungha Shin, Ehn-Kyoung Choi, Youngjin Choi, Ja-Young Jang, Jihyun Kim, Heon-Sang Jeong, Wooryoung Lee, Yoon-Bok Lee, Seung Up Kim, Seong Soo Joo, Yun-Bae Kim

**Affiliations:** ^1^College of Veterinary Medicine, Chungbuk National University, Cheongju, Chungbuk 362-763, Republic of Korea; ^2^Ajou University School of Medicine, Suwon, Gyeonggi 443-380, Republic of Korea; ^3^Department of Food Science and Technology, Chungbuk National University, Cheongju, Chungbuk 362-763, Republic of Korea; ^4^Department of Pediatrics, Soonchunhyang University Hospital, Seoul 140-743, Republic of Korea; ^5^Central Research Institute, Dr. Chung's Food Co. Ltd., Cheongju, Chungbuk 361-782, Republic of Korea; ^6^Division of Neurology, University of British Columbia Hospital, Vancouver, BC, Canada V6T 2B5; ^7^Department of Marine Molecular Biotechnology, Gangneung-Wonju National University, Gangneung, Gangwon 210-702, Republic of Korea

## Abstract

*Objective*. Since oligodendrocyte progenitor cells (OPCs) are the target cells of neonatal hypoxic-ischemic encephalopathy (HIE), the present study was aimed at investigating the protective effects of *N*-acetyl-l-cysteine (NAC), a well-known antioxidant and precursor of glutathione, in OPCs as well as in neonatal rats. *Methods*. In *in vitro* study, protective effects of NAC on KCN cytotoxicity in F3.Olig2 OPCs were investigated via MTT assay and apoptotic signal analysis. In *in vivo* study, NAC was administered to rats with HIE induced by hypoxia-ischemia surgery at postnatal day 7, and their motor functions and white matter demyelination were analyzed. *Results*. NAC decreased KCN cytotoxicity in F3.Olig2 cells and especially suppressed apoptosis by regulating Bcl2 and p-ERK. Administration of NAC recovered motor functions such as the using ratio of forelimb contralateral to the injured brain, locomotor activity, and rotarod performance of neonatal HIE animals. It was also confirmed that NAC attenuated demyelination in the corpus callosum, a white matter region vulnerable to HIE. *Conclusion*. The results indicate that NAC exerts neuroprotective effects *in vitro* and *in vivo* by preserving OPCs, via regulation of antiapoptotic signaling, and that F3.Olig2 human OPCs could be a good tool for screening of candidates for demyelinating diseases.

## 1. Introduction

Cerebral palsy (CP) resulting from hypoxic-ischemic encephalopathy (HIE) is one of the most devastating neurological diseases in children exhibiting diverse neurobehavioral symptoms including motor, perceptual, visual, behavioral, and cognitive disorders [1, 2]. HIE during delivery and/or intrauterine infection has been reported to be an important feature in many cases with white matter injury (WMI) showing periventricular leukomalacia (PVL) [1, 3]. Oligodendrocyte progenitor cells (OPCs) and immature oligodendrocytes were found to be particularly susceptible to free radicals and inflammatory cytokines induced by hypoxic-ischemic insults [1, 2, 4]. Therefore, loss of OPCs and immature oligodendrocytes during their maturation process of the central nervous system (CNS) is a key feature of HIE [1, 4].

Earlier, we established a human OPC line (HB1.F3.Olig2) by transducing HB1.F3 human neural stem cell (NSC) line with a retroviral vector encoding* Olig2*, an essential regulator of oligodendrocyte development, and confirmed the characteristics for the oligodendrocyte lineage cells [5, 6]. Since OPCs are the target cells of HIE [1, 2, 4], we hypothesized that compounds displaying protective activity against hypoxic cytotoxicity of OPCs would be more effective in HIE animal models than those screened from neuronal culture.

In the treatment of HIE, current therapeutic strategies are very limited and restricted to supportive intensive care such as clinical hypothermia [7–9]. Recently, experimental administration of antioxidative, anti-inflammatory, and neuroprotective compounds such as vitamin C (ascorbic acid),* N*-acetyl-l-cysteine (NAC), minocycline, and erythropoietin was found to attenuate WMI and delay the progress of physical dysfunction [10]. However, there are controversial results on the effects of vitamin C, minocycline, and erythropoietin in animal models or in humans [11–13].

Vitamin C and NAC are well-known broad spectrum antioxidants used as drugs and functional heath foods. NAC, an antidote of acetaminophen overdose, especially has been proposed as a candidate for treatment of vascular and nonvascular neurological disorders [14]. Indeed, it was reported that NAC prevented the decrease in myelin-related gene expression of primary oligodendrocytes exposed to cytokines [15], which was focused on multiple sclerosis (MS), another adult demyelinating disease, using mature oligodendrocytes. Paintlia et al. [16] demonstrated that NAC treatment attenuated lipopolysaccharide- (LPS-) induced degeneration of OPCs in developing rat brain. Although there are no clear differences in the pathogenic mechanisms and factors between CPs caused by hypoxia during delivery and intrauterine infection, their study is mainly describing anti-inflammatory activity of NAC. In the present study, we assessed the protective effects of NAC and vitamin C against the cytotoxicity of potassium cyanide (KCN), a well-known hypoxia-inducing neurotoxin [17] as a model of hypoxic OPCs damage.

As animal models for HIE, ischemia (carotid artery ligation) followed by hypoxia (8% O_2_ for 2 h) caused demyelination and neurobehavioral abnormalities in rats [18–20]. In order to confirm our hypothesis, we assessed* in vitro* cytoprotective effects of NAC in F3.Olig2 human OPCs as well as the neuroprotective activities of NAC in HI animals.

## 2. Materials and Methods

### 2.1. F3.Olig2 Human OPCs

An immortalized NSC line (F3) was established from primary cultures of a 15-week gestational human fetal brain by infecting with a retroviral vector encoding v-*myc* oncogene [21]. The Clinical Research Screening Committee involving Human Subjects of the University of British Columbia (Ethics Committee) approved the use of the fetal tissue, and the fetal tissues were obtained from the Anatomical Pathology Department of Vancouver General Hospital. Subsequently, F3.Olig2 cells were obtained by transducing F3 NSCs with a retroviral vector encoding* Olig2*. The characteristics of F3.Olig2 cells as oligodendrocyte lineage cells were confirmed with immunoreactivity to O4 and CNPase, specific markers for OPCs [5, 6].

### 2.2. MTT Assay for KCN Cytotoxicity

The protective effects of NAC and vitamin C against KCN cytotoxicity were determined via 3-(4,5-dimethylthiazol-2-yl)-2,5-diphenyl-tetrazolium bromide (MTT) assay. Briefly, F3.Olig2 cells (1 × 10^6^ cells/mL) were seeded in each well containing 100 *μ*L of Dulbecco's modified Eagle's medium (DMEM) supplemented with 10% fetal bovine serum, 1% d-glutamine, 100 *μ*g/mL gentamicin, and 2.5 U/mL amphotericin B in a 96-well plate. After 2 h incubation, the cells were treated with various concentrations (0.25–7.5 mM) of NAC or vitamin C, followed immediately by KCN (5 mM). After 18 h incubation and washing twice with fresh medium, 10 *μ*L of MTT (5 mg/mL DMEM, filtered) was added and incubated for additional 2 h. The medium was discarded, and the formazan blue formed in the living cells was dissolved with 50 *μ*L of dimethyl sulfoxide. The optical density was measured at 570 nm in 30 min using an ELISA reader (Molecular Devices, Sunnyvale, CA, USA). The experiments were performed 5 times, and mean values were presented.

### 2.3. Flow Cytometric Analysis of Annexin V

In order to investigate antiapoptotic effects, F3.Olig2 cells (2 × 10^6^) were treated with various concentrations of NAC (0.5–5 mM) or vitamin C (0.15 mM), followed by KCN (5 mM) for 18 h. Induction of apoptosis was measured by flow cytometry using an Annexin V-FITC/PI double staining kit according to the manufacturer's indications (Enzo Life Sciences, Lörrach, Germany). In brief, cells (1 × 10^6^ cells/mL) were suspended in 1x binding buffer, and Annexin V (1 *μ*L) and propidium iodide (PI, 2.5 *μ*L) were added to 96 *μ*L cell suspension. The mixture was kept on ice for 10 min in the dark and analyzed by FACScan (Becton Dickinson, Mountain View, CA, USA). The experiments were performed 5 times, and representative analytical data and mean values were presented.

### 2.4. Western Blot Analysis of Apoptosis-Related Molecules

F3.Olig2 cells were lysed in 1% RIPA buffer containing protease and phosphatase inhibitors (Roche, Mannheim, Germany) and whole cell lysates were separated by 10% SDS-PAGE. After electrophoresis, proteins were transferred onto polyvinylidene fluoride membranes and the membranes were blocked with 5% skim milk in Tris-buffered saline solution containing 0.1% Tween-20. The membrane was then immunoblotted with primary antibodies such as anti-Bcl2, anti-Bad, anti-extracellular signal-regulated protein kinase (ERK), anti-phosphor-ERK (p-ERK), and anti-glyceraldehyde 3-phosphate dehydrogenase (GAPDH) (Santa Cruz Biotechnology, Santa Cruz, CA, USA), which was followed by incubation with horseradish peroxidase-conjugated anti-rabbit or anti-mouse secondary antibodies (Cell Signaling Technology, Beverly, MA, USA). Blots were developed using an ECL solution and exposed to X-ray film (Amersham Bioscience, Piscataway, NJ, USA). The experiments were performed 5 times, and representative bands and mean densities normalized to GAPDH were presented.

### 2.5. Hypoxic-Ischemic Encephalopathy Animal Model and Treatment

Pregnant Sprague-Dawley rats were purchased from Daehan-Biolink (Eumseong, Chungbuk, Korea). The animals (*n* = 7/group) were maintained at a constant temperature (23 ± 2°C), relative humidity of 55 ± 10%, and 12 h light/dark cycle and fed with standard rodent chow and purified water* ad libitum*. Neonates were obtained from natural delivery, and male pups of postnatal day (PND) 7 underwent HI; that is, their left common carotid artery was occluded and placed in an 8% oxygen/92% nitrogen incubator (36°C) for 2 h [19, 20]. The rats were intraperitoneally administered with NAC (100 mg/kg) 30 min prior to the carotid artery occlusion surgery at PND 7 and once a day to PND 44. Control animals only underwent the Sham operation and vehicle treatment. All experimental procedures were approved and carried out in accordance with the Institutional Animal Care and Use Committee of Laboratory Animal Research Center at Chungbuk National University, Cheongju, Chungbuk, Korea.

### 2.6. Measurement of Neurobehavioral Functions

#### 2.6.1. Cylinder Test

For the evaluation of forelimb use asymmetry by brain damage, the ratio of right (impaired) forelimb (contralateral to left carotid artery occlusion) use was analyzed at PND 20, 30, and 40 [20]. Each animal was individually placed in a glass cylinder (21 cm in diameter, 34 cm in height) for 3 min. The weight-bearing forepaw to contact the cylinder wall during a full rear was recorded as left (normal), right (impaired), or both. Paw preference was calculated as ((normal forepaw − impaired forepaw)/(normal forepaw + impaired forepaw + both) × 100%).

#### 2.6.2. Locomotor Activity

Spontaneous activities and exploratory behaviors were evaluated using a video tracking system (Smart v2.5; Panlab Technology, Barcelona, Spain), connected to a CCTV (Samsung, Changwon, Gyeongnam, Korea) at PND 20, 30, and 40 [20, 22]. Rats were placed in a quiet chamber with a dim light. The types of movement, that is, resting, slow-moving, and fast-moving times, were recorded for 5 min, and the ratio was analyzed.

#### 2.6.3. Rotarod Performance

Motor balance and coordination were evaluated using a rotarod test system (Panlab Technology) at PND 20, 30, and 40 [20]. Rats were placed on a rotating rod at a constant speed of 12 rpm, and the time it took for the rats to fall off the rod was recorded. The average latency was calculated from 3 consecutive measurements.

### 2.7. Immunohistochemistry in Brain Sections

In order to confirm the integrity of host myelin at PND 45, the rat brain was perfusion-fixed with 10% paraformaldehyde solution and postfixed in the same solution for 48 h, followed by cryoprotection in 30% sucrose for 72 h. Paraffin-embedded sections were stained with Luxol fast blue (LFB) for examination of myelins. Coronal cryosections in 30 *μ*m thickness were prepared and processed for immunostaining for myelin basic protein (MBP). Brain sections were incubated with primary antibody specific for MBP (1 : 200; rabbit polyclonal, Chemicon, Temecula, CA, USA) overnight at 4°C, followed by Alexa Fluor 594-conjugated anti-rabbit IgG (1 : 1,000; Molecular Probes, Eugene, OR, USA) for 2 h at room temperature [20]. All samples were evaluated immediately after staining and photographed with a laser-scanning confocal microscope (LSM710; Zeiss, New York, NY, USA). In order to quantify the immunoreactivity of host MBP, the photographs were analyzed with a Digital Image Analyzer (Image Inside; Focus, Seoul, Korea) for the red intensity, and expressed as a % of the control (normal) group.

### 2.8. Statistical Analysis

Data are presented as mean ± standard error. The statistical significance was determined by one-way analysis of variance, followed by post hoc Tukey's multiple-comparison test. *P* values < 0.05 were considered to be statistically significant.

## 3. Results

**Figure 1:**
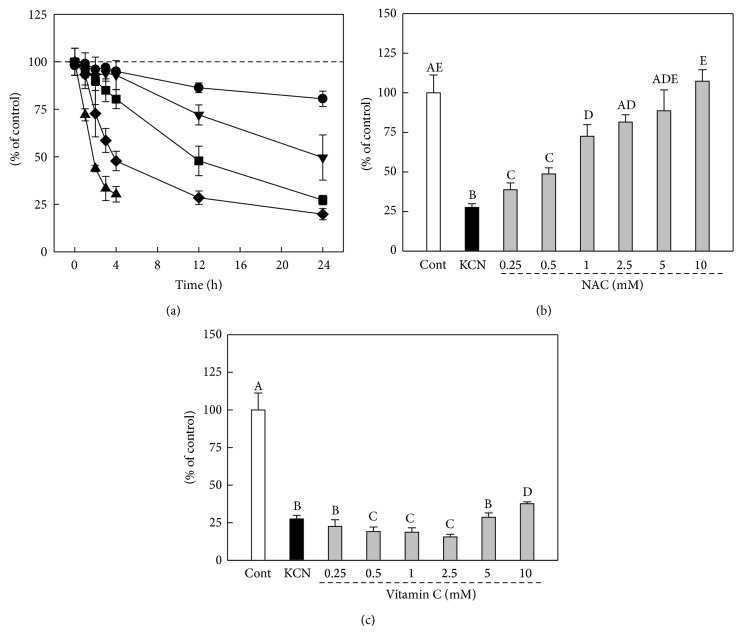
Effects of NAC and vitamin C on KCN cytotoxicity in F3.Olig2 human oligodendrocyte progenitor cells. (a) Cytotoxicity of KCN in MTT assay. F3.olig2 cells were with various concentrations (●: 0.5 mM, ▼: 1 mM, ■: 2.5 mM, ◆: 5 mM, and ▲: 7.5 mM) of KCN for 18 h. (b, c) F3.Olig2 cells were treated with KCN (5 mM) alone or in combination with NAC (b) or vitamin C (c) and assayed after 18 h incubation. Values with different superscript letters represent a significant difference, *P* < 0.05.

KCN induced cell death of F3.Olig2 OPCs in a concentration- and time-dependent manner as determined by* in vitro* MTT assay. KCN induced mortalities of 52.2, 71.5, and 80.2% at 4, 12, and 24 h with 5 mM KCN, respectively (Figure 1(a)). We selected 18 h exposure to 5 mM KCN to assess the protective effects of NAC over KCN cytotoxicity. NAC (0.25–10 mM) significantly reversed the cytotoxicity of KCN in a concentration-dependent manner, leading to 72.5% survivability of the cells at 1 mM, compared to 27.6% in vehicle-treated cells (Figure 1(b)). In contrast, vitamin C did not show a protective activity on the KCN cytotoxicity, further decreasing the cell survivability at 0.5–2.5 mM (Figure 1(c)).

**Figure 2:**
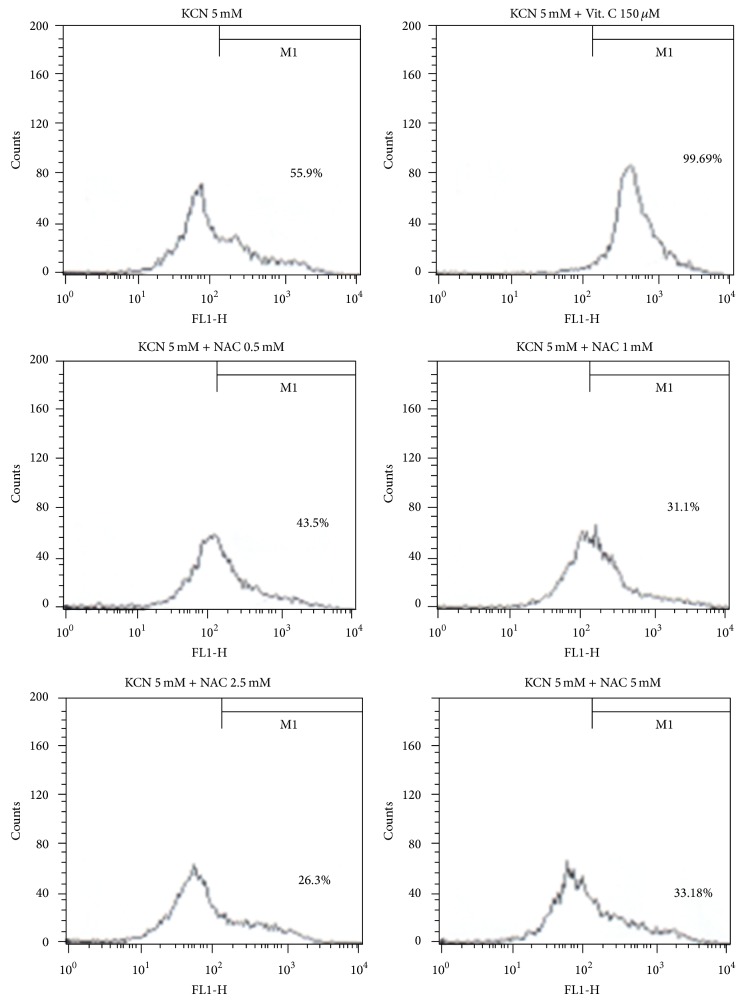
Representative flow cytometric analysis of Annexin V/PI in F3.Olig2 human oligodendrocyte progenitor cells. F3.Olig2 cells were treated with KCN (5 mM) alone or in combination with vitamin C or NAC for 18 h.

Exposure to KCN (5 mM) caused apoptotic death of F3.Olig2 OPCs, leading to 56.8% Annexin V/PI double positivity (Figure 2, Table 1). Notably, NAC at 0.5–2.5 mM decreased the ratio of apoptotic cells in a concentration-dependent manner, although a higher concentration (5 mM) did not further decrease the ratio. In contrast, vitamin C even a low concentration (0.15 mM) significantly elevated cell death, indicative of an enhanced apoptosis similarly in MTT assay.

**Figure 3:**
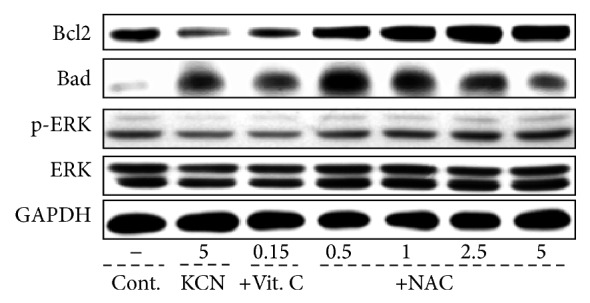
Representative western blot analysis of apoptosis-related proteins in F3.Olig2 human oligodendrocyte progenitor cells. F3.Olig2 cells were treated with KCN (5 mM) alone or in combination with vitamin C or NAC for 18 h.

In order to elucidate the change in apoptosis-related signals, we analyzed the expression of Bcl2, Bad, ERK, and p-ERK in F3.Olig2 cells. KCN markedly increased the expression of Bad, a proapoptotic molecule, while it decreased Bcl2 and p-ERK, the antiapoptotic proteins (Figure 3). NAC (0.5–5 mM) upregulated the expression of Bcl2 and p-ERK in a concentration-dependent manner, to levels higher than in normal cells at 1–5 mM, while vitamin C (0.15 mM) did not affect the expression of signaling molecules in F3.Olig2 OPCs following KCN exposure (Table 1).

**Figure 4:**
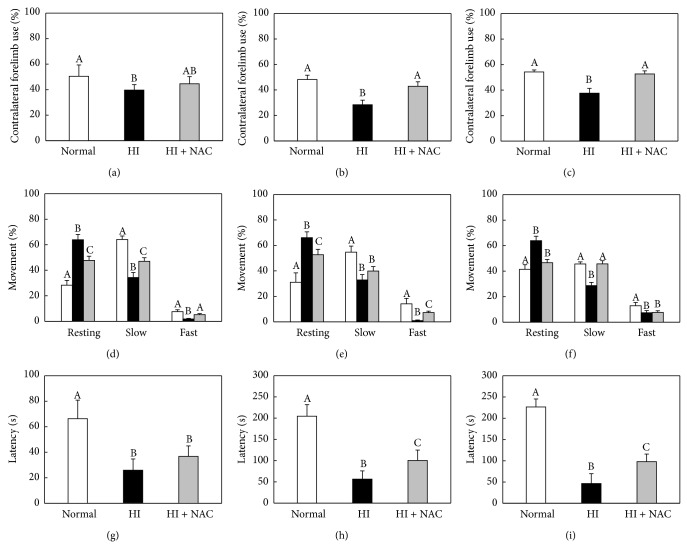
Recovery of motor functions of HI rats by NAC. Rats were subjected to hypoxic-ischemic (HI) surgery at postnatal day (PND) 7, and behavioral functions in cylinder test (a–c), locomotor activity (d–f), and rotarod performance (g–i) were assessed at PND 20 (a, d, and g), 30 (b, e, and h), and 40 (c, f, and i). White: normal animals, black: HI alone, and gray: HI + NAC. Values with different superscript letters represent a significant difference, *P* < 0.05.

Based on the different effects of NAC and vitamin C on the KCN cytotoxicity in F3.Olig2 cells, we selected NAC as a candidate compound for the treatment of HI model animals. In the cylinder test, normal animals used their left and right forelimbs in similar ratios (50 : 50%) at PND 20, 30, and 40 (Figures 4(a)–4(c)). However, rats subjected to HI at PND 7 showed significantly decreased (<40%) use of the contralateral forelimb at PND 20, 30, and 40. Such a reduced use of the contralateral forelimb was near fully recovered at PND 30 and 40 by daily intraperitoneal administration of NAC (100 mg/kg) from PND 7.

Normal animals exhibited active movement in locomotor activity analysis, in which the sum (60–70%) of slow-moving and fast-moving times was longer than resting time (30–40%) at all PND 20–40 (Figures 4(d)–4(f)). However, the resting time greatly increased in HI rats (>65%), leading to significant decreases in moving times from PND 20 to PND 40. By comparison, treatment with NAC markedly recovered the HI-induced decrease in global activity.

HI caused impairment of motor coordination in rotarod performance, leading to a marked reduction in the latency time by 60–80% at PND 20–40 (Figures 4(g)–4(i)). Interestingly, however, such decreased rotarod performances were significantly improved at PND 30 and PND 40 by NAC treatment.

**Figure 5:**
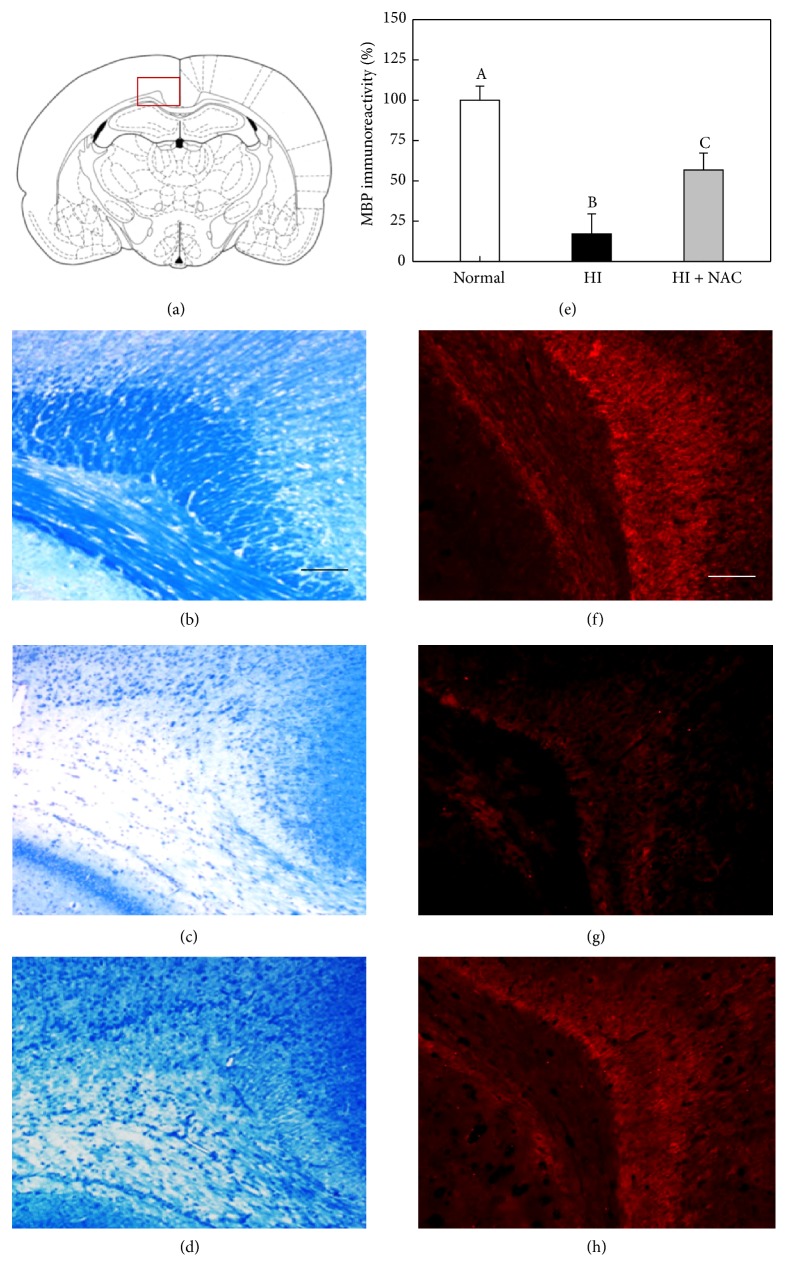
Recovery of LFB-stained myelins and MBP immunoreactivity in rat corpus callosum by NAC. Integrity of myelins was analyzed with LFB staining and MBP immunoreactivity at postnatal day 45. The square box in (a) represents an examined area of corpus callosum. The brain tissues were stained with LFB (b–d) or MBP antibody (f–h). (b, f) Normal animals, (c, g) HI alone, and (d, h) HI + NAC. Scale bars = 500 *μ*m. Values with different superscript letters in (e) represent a significant difference in MBP immunoreactivity from (f–h), *P* < 0.05.

Normal animals exhibited a good integrity of myelins in the corpus callosum, a white matter region vulnerable to HI injury, revealing strong LFB-staining intensity as well as MBP immunoreaction (Figures 5(b) and 5(f)) [20]. By comparison, the LFB-staining intensity and MBP immunoreactivity were markedly reduced in HI animals as observed at PND 45, indicative of a severe demyelination and loss of host oligodendrocytes (Figures 5(c), 5(g), and 5(e)). Notably, however, the HI-induced loss of myelins and MBP was significantly attenuated by NAC treatment (Figures 5(d), 5(h), and 5(e)).

## 4. Discussion

It is well known that damage of OPCs and preoligodendrocytes during the maturation process of the CNS is a key factor for HIE, and the loss of OPCs and hypomyelination of the CNS due to perinatal HI or intrauterine infection are major features of PVL [1, 2, 4]. Although there have been studies on drug screening* in vitro* using primary oligodendrocytes [15] or neuronal cell lines such as PC12 cells [17], here we suggest human F3.Olig2 OPCs as an appropriate cell system to screen new drug candidates for treatment of PVL.

Among diverse candidate compounds [10], vitamin C has been suggested to be neuroprotective in a neonatal HI model [23]. However, vitamin C is known to act as a prooxidant in the presence of transition metal ions and was shown to cause neurotoxicity* in vitro* in rodent cortical neurons [24]. In addition to the cytotoxicity in cortical neurons, aggravating effects of vitamin C on the cytotoxicity and apoptosis induced by KCN were observed in F3.Olig2 OPCs. In fact, there are controversial results on the effects of vitamin C in human CP patients [11].

In previous studies, NAC exerted anti-inflammatory and antioxidative potentials in the diverse CNS diseases [25–30]. In cases of multiple sclerosis and traumatic brain injury, NAC suppressed TNF-*α*-mediated via downregulating the activation of nuclear factor-*κ*B (NF-*κ*B) [25, 26]. Also, in the neurodegenerative diseases such as amyotrophic lateral sclerosis (ALS), Parkinson disease (PD), Huntington disease (HD), and Alzheimer disease (AD), NAC reduced tissue injury and endothelial cell apoptosis by enhancing antioxidant enzymes and/or inhibiting lipid peroxidation [25, 27–29]. NAC also improved microcirculation and tissue oxygenation that may facilitate tissue regeneration in the degenerative diseases [30]. Accordingly, NAC has attracted investigators' attention for its efficacy in diverse vascular and nonvascular neurological disorders [14] including both intrauterine infection and HI models of PVL [9, 10]. In fact, NAC suppressed inflammatory cytokines in amniotic fluid and placenta in LPS-treated rats [31] and displayed neuroprotective activity in LPS-sensitized rat pups exposed to HI [32]. In addition, NAC improved platelet aggregation and hemodynamics in the brain of piglets with hypoxia-reperfusion (IR) injury [33, 34]. Notably, NAC enhanced the efficacy of hypothermia in reducing infarct volume after focal HI brain injury in neonatal rats [35].

Interestingly, NAC prevented endotoxin-induced degeneration of OPCs and hypomyelination in developing rat brain in an intrauterine infection model of PVL [16]. In the present study, we demonstrated neuroprotective effects of NAC both* in vitro* and* in vivo*; that is, NAC prevented KCN-induced cytotoxicity of F3.Olig2 human OPCs and hypomyelination in developing rat brain in an HI model of PVL. Although more detailed studies on the mechanism(s) of NAC for the preservation of myelins* in vivo* remained to be clarified, it is proposed that NAC may protect host OPCs against hypoxic damage as confirmed in* in vitro* study. In contrast to the aggravating effects of vitamin C, NAC decreased KCN cytotoxicity; that is, NAC preserved F3.Olig2 cell viability impaired by KCN as measured by MTT assay. It is believed that such a protective effect of NAC might be in part due to the regulatory activity related to survival including Bcl2 and p-ERK. NAC upregulated Bcl2/p-ERK prosurvival proteins in a concentration-dependent manner and downregulated Bad proapoptotic protein at a high concentration (5 mM), which may led to a higher survivability of KCN-exposed F3.Olig2 human OPCs. However, is it suggested that NAC preserves mitochondrial function and blocks apoptotic signaling at different concentration ranges, as seen in MTT and Annexin V analyses, respectively.

In spite of extensive studies on the candidate therapeutics for PVL, there are very limited clinical studies on the efficacy of chemical drugs in preterm or perinatal PVL patients. In a randomized clinical trial of preterm newborns, continuous intravenous infusion of NAC (16–32 mg/kg) for 6 days after birth reduced the incidence of PVL, although this was a very preliminary study [36]. It is worthy to note that hypothermia plus NAC combination therapy improves infarct volume, myelin expression, and functional outcomes after focal HI injury in neonatal rats [35].

It should be emphasized that timely delivery of neuroprotective treatment is the most important in neonates suffering from hypoxic injury or intrauterine infection [9]. In a piglet IR model, intravenous injection of NAC (150 mg/kg bolus and 20 or 100 mg/kg/h for 24 h) improved hemodynamics and neural oxidative stress, in addition to platelet aggregation [33, 34]. In the present study, daily intraperitoneal injection of NAC (100 mg/kg) recovered neurobehavioral dysfunction in neonatal HI rats, along with the neuroprotective effect preserving a part of MBP reactivity in the corpus callosum. For an enhanced efficacy of NAC, a dose-range-finding study with diverse administration routes including continuous intravenous infusion is required. Moreover, it is expected that the optimized treatment of NAC in combination with hypothermia or other neuroprotectants could be a promising strategy for the effective intensive care of PVL patients.

To date, there are no effective regimens for the prevention of HIE in CP patients. In the present study, we used F3.Olig2 human OPCs for the first time to screen candidate compounds for PVL. We showed protective effects of NAC, but aggravating potential of vitamin C on KCN cytotoxicity in the F3.Olig2 cells. Furthermore, NAC recovered motor function of HI rats by attenuating the loss of myelins and MBP in the corpus callosum. Therefore, it is suggested that F3.Olig2 human OPCs could serve as a good cell system for the screening of demyelinating diseases and that NAC could be a good candidate for the treatment for HI brain injury.

## Figures and Tables

**Table 1:** Statistical analysis of the apoptosis-related molecules from Figures 2 and 3.

Treatment (mM)	Annexin V positivity (%)	Bcl2 (relative to GAPDH)	Bad (relative to GAPDH)	p-ERK (relative to GAPDH)
Vehicle	3.6 ± 2.7^a^	0.28 ± 0.13^a^	0.001 ± 0.001^a^	0.097 ± 0.015^a^
KCN (5.0)	56.8 ± 4.8^b^	0.11 ± 0.06^b^	0.312 ± 0.124^b^	0.065 ± 0.011^b^
+vitamin C (0.15)	98.6 ± 7.4^c^	0.21 ± 0.11^ab^	0.282 ± 0.152^b^	0.068 ± 0.082^ab^
+NAC (0.5)	42.7 ± 3.4^d^	0.37 ± 0.14^ac^	0.521 ± 0.206^b^	0.124 ± 0.113^a^
+NAC (1.0)	32.3 ± 5.1^df^	0.68 ± 0.20^d^	0.341 ± 0.093^b^	0.156 ± 0.108^c^
+NAC (2.5)	26.1 ± 2.8^e^	0.91 ± 0.26^d^	0.238 ± 0.062^b^	0.202 ± 0.077^c^
+NAC (5.0)	31.3 ± 4.2^ef^	0.82 ± 0.22^d^	0.110 ± 0.046^c^	0.235 ± 0.086^c^

Values (*n* = 5) in the same column with different superscript letters represent a significant difference, *P* < 0.05.
